# Identification and validation of a novel cuproptosis-related signature as a prognostic model for lung adenocarcinoma

**DOI:** 10.3389/fendo.2022.963220

**Published:** 2022-10-24

**Authors:** Yuqiao Chen, Lu Tang, Wentao Huang, Youyu Zhang, Fakolade Hannah Abisola, Linfeng Li

**Affiliations:** ^1^ Department of Thoracic Surgery, Xiangya Hospital, Central South University, Changsha, Hunan, China; ^2^ Department of Anesthesiology, Xiangya Hospital, Central South University, Changsha, Hunan, China; ^3^ National Clinical Research Center for Geriatric Disorders, Changsha, Hunan, China; ^4^ Xiangya Lung Cancer Center, Xiangya Hospital, Central South University, Changsha, Hunan, China

**Keywords:** lung cancer, cuproptosis, prognostic signature, bioinformatic analysis, overall survival

## Abstract

**Background:**

Cuproptosis is a novel form of copper-induced cell death that targets lipoylated tricarboxylic acid (TCA) cycle proteins. However, its prognostic role in lung adenocarcinoma (LUAD) remains unclear. This study aimed to establish a cuproptosis-related prognostic signature for patients with LUAD.

**Methods:**

Transcriptome data of LUAD samples were extracted from the Cancer Genome Atlas (TCGA) and Gene Expression Omnibus (GEO) databases. The prognostic value of cuproptosis-related genes (CRGs) was investigated using Cox regression analysis to develop a cuproptosis-related prognostic model. Kyoto Encyclopedia of Genes and Genomes (KEGG), gene ontology (GO) and gene set variation analysis (GSVA) were conducted to characterize different biological activities or pathways between high- or low-CRG groups. The expression pattern and prognostic values of CRGs were validated in 37 paired tumor–normal samples using quantitative PCR. Furthermore, *in vitro* experiments were performed to investigate the relationship between cuproptosis and CRG expression and to explore the function of target genes in cuproptosis.

**Results:**

Among the 36 CRGs, 17 genes were upregulated, and 3 genes were downregulated in LUAD. A total of 385 CRGs were identified using Pearson correlation analysis. A cuproptosis-related signature was constructed using least absolute shrinkage and selection operator (LASSO) analysis. The prognostic value of the cuproptosis-related signature was validated in six external validation cohorts and in LUAD specimens from our facility. Patients in the high-risk group based on the CRG signature score had shorter overall survival than those in the low-risk group in both the datasets and clinical specimens. *In vitro* experiments revealed that the expression of *BARX1*, *GFRA3*, and *KHDRBS2* was upregulated after cuproptosis was induced by elesclomol–CuCL_2_, whereas the upregulation was suppressed on pretreatment with tetrathiomolybdate (TTM), a chelator of copper. Further, the cell proliferation assay revealed that the *BARX1* and *GFRA3* deficiency facilities the cuproptosis induced by elesclomol–CuCL_2_.

**Conclusion:**

This study established a new CRG signature that can be used to predict the OS of LUAD patients. Moreover, the knockdown of *BARX1* and *GFRA3* could increase the sensitivity of LUAD cells to the cuproptosis.

## Introduction

Lung cancer is the leading cause of cancer-related deaths worldwide, accounting for nearly 1.3 million deaths per year. More than half of lung cancer patients have metastatic disease. Metastatic lung cancer has a 5-year survival rate of only 6% (1). Lung adenocarcinoma (LUAD) is the most common type of lung cancer and accounts for 50% of all cases. The molecules and pathways mediating the occurrence and progression of LUAD require further investigation. With the popularity of lung cancer screening and introduction of tyrosine kinase inhibitors (TKI), the survival rate of LUAD patients has improved to some extent (2). However, there is a high risk of acquired TKI drug resistance and metastatic relapse after excision (3). Therefore, it is critical to identify reliable and promising prognostic biomarkers for patients with LUAD.

Copper (Cu) is an essential mineral nutrient for all living organisms and is a fundamental element in many biological processes, including mitochondrial respiration, iron uptake, and antioxidant and detoxification processes (4). Cancer cells have a higher demand for Cu than do normal cells. Some studies have shown elevated Cu concentrations in tumors or serum from patients with a variety of cancers, including breast, lung, gastrointestinal, oral, thyroid, gallbladder, gynecological, and prostate cancers (5). Also, some evidence exists that Cu may play a role in the initiation and progression of cancer. Additionally, Cu promotes angiogenesis, which is essential for tumor progression and metastasis (6–9). However, when the concentration of Cu exceeds a threshold set by conserved homeostatic systems, it becomes toxic. Tsvetkov et al. recently presented copper-induced cell death known as cuproptosis, which targets lipoylated tricarboxylic acid (TCA) cycle proteins (10). This type of Cu toxicity refers to a previously unknown cell death mechanism that differs from all other known cell death mechanisms, such as apoptosis, iron death, pyroptosis, and necroptosis.

Cuproptosis is primarily caused by Ferredoxin 1 (*FDX1*)-mediated mitochondrial proteotoxic stress when Cu levels increase. On the one hand, *FDX1* converts Cu^2+^ to Cu^+^, resulting in lipoylation and aggregation of enzymes (particularly *DLAT*) involved in mitochondrial TCA cycle control. *FDX1*, on the other hand, causes the Fe–S cluster proteins to become unstable. Cu importers (e.g., *SLC31A1*) and exporters (e.g., *ATP7B*) influence cuproptosis sensitivity by altering intracellular Cu^+^ levels. Glutathione (GSH) inhibits cuproptosis by acting as a thiol-containing Cu chelator, whereas buthionine sulfoximine (BSO) promotes apoptosis by depleting GSH. UK5099, a mitochondrial pyruvate carrier (MPC) inhibitor, and ETC complex I/III inhibitors (such as rotenone and antimycin A) can reduce elesclomol-induced cuproptosis (11). Cuproptosis is expected to be developed as a key target for cancer treatment.

In the present study, CRGs were identified using Pearson correlation analysis. A cuproptosis-related signature was constructed using least absolute shrinkage and selection operator (LASSO) analysis. The prognostic value of the cuproptosis-related signature was validated in six external validation cohorts. Then, clinical samples were used to validate the expression patterns and prognostic value of CRGs. Finally, *in vitro* experiments were performed to investigate the role and biological activities of CRGs in cuproptosis.

## Materials and methods

### Data collection and identification of CRGs

Transcriptome data and clinical information from the Cancer Genome Atlas (TCGA)-LUAD were downloaded from UCSC XENA (https://xenabrowser.net/). Totally, 36 CRGs were collected from a previous study (10) and are listed in the **Supplementary Table S1**. The Pearson correlation coefficient was then calculated to define the CRGs. The analyzed genes were identified as CRGs if the *p*-value was less than 0.001 and the absolute value of the Pearson correlation coefficient was greater than 0.3 (|R|>0.3).

### The development and validation of a prognostic cuproptosis-related signature

A total of 506 patients from the TCGA LUAD dataset were randomly separated into training(N=253) and validation(N=253) cohorts in a 1:1 ratio. First, using univariate Cox regression, CRGs with prognostic value were identified in the training cohort. In addition, based on the CRG expression and survival data, a prognostic gene signature was established using the least absolute shrinkage and selection operator (LASSO) Cox regression analysis with the R package “glmnet.” The risk score of this signature was calculated as follows: risk score = coefficient1×expRNA1 + coefficient2×expRNA2 + coefficient3×expRNA3 + …+ coefficient(n)×expRNA(n).

The inclusion criteria of the GEO data were as follows (1): datasets including LUAD samples; (2) datasets with RNA-seq or gene microarrays; (3) datasets with clinical survival information. The exclusion criteria of the GEO datasets were the following: (1) datasets exploring the other tumors that are not LUAD; (2) no survival data in the dataset; (3) methylation data or miRNA microarrays or the other datatype. Finally, Six external cohorts downloaded from the Gene Expression Omnibus (GEO) database were used to further validate the prognostic efficacy of the CRG signature, including 116 samples of GSE36471 (12), 158 samples of GSE31210 (13), 180 samples of GSE42127 (14), 442 samples of GSE72094 (15), 90 samples of GSE11969 (16), and 462 samples of GSE68465 (17).

### The construction of a prognostic nomogram

The association between independent risk factors and prognosis was evaluated using a univariate Cox regression analysis. Multivariate Cox regression analysis was used to determine if the risk scores and clinical parameters were independent predictors of overall survival (OS). Risk score and other clinical indications were used to create a nomogram that predicted the 1-, 3-, and 5-year OS of patients with LUAD. To determine the predictive accuracy of the nomogram, we employed data calibration curves that were created to ascertain whether the predicted and observed OS probabilities were in agreement. The concordance index (C-index) was calculated to scale the nomogram’s ability to predict and discriminate prognosis. The C-index ranged from 0.5–1.0; higher the C-index, better the distinguishing ability of the predictive model.

### Functional enrichment analysis and tumor-infiltrating immune cells estimation

Gene Ontology (GO) analysis was performed on the differentially expressed genes (DEGs) from the high-risk and low-risk groups to determine the biological processes (BP), molecular functions (MF), and cellular components (CC) associated with the cuproptosis-related signature. The signaling pathways associated with the cuproptosis-related signature were identified using Kyoto Encyclopedia of Genes and Genomes (KEGG) pathway analysis.

Gene set variation analysis (GSVA) (18) was further used to explore the pathways involved in the DEGs in different risk groups. The Molecular Signatures Database (MSigDB) v.5.2 (http://software.broadinstitute.org/gsea/msigdb/index.jsp) was used to get gene sets for board hallmarkers.

The algorithm of “ssGSEA”, described by Charoentong et al. (19), was used to quantify tumor infiltration immune cells from the transcriptome data based on specific molecular markers.

### Clinical specimens and quantitative real-time PCR

The CRG expression profiles were tested in paired tumor–normal samples from 37 patients with LUAD who underwent lobectomy between January 2015 and June 2020 at Xiangya Hospital, Central South University. In addition, the prognostic value of the CRG signatures was evaluated. The ethics committee of the Xiangya Hospital of Central South University approved this study. Written informed consent was obtained from all patients involved in this study.

An RNA isolator was used to extract total RNA from LUAD and normal tissues (Vazyme, Nanjing, China). The NovoScript^®^ Plus All-in-one 1st Strand cDNA Synthesis SuperMix Kit with gDNA (genomic DNA) was used to generate complementary DNA (cDNA) from 1 ng of total RNA (Novoprotein, Jiangsu, China). Real-time quantitative PCR (qPCR) was performed on a QuantStudio5 Real-Time PCR System (Applied Biosystems, USA) using the Hieff^®^ qPCR SYBR Green Master Mix (Yeasen, Shanghai, China). The relative expression levels of the putative CRGs were normalized to those of endogenous ACTB. Primers listed in **Supplementary Table S2** were synthesized by Tsingke Biotechnology Co., Beijing, China.

### Cuproptosis-induced model for testing the expression of cuproptosis related genes

The LUAD cell line PC9 was chosen for *in vitro* experiments. PC9 cells were cultured in DMEM culture medium (Servicebio, Wuhan, China), supplemented with 10% Fetal Bovine Serum (Biological Industries, Beijing, China) in a standard humidified incubator with 5% CO2 at 37°C. The PC9 cell line was tested by short tandem repeat (STR) analysis and authenticated by Center for Genetic Genomic Analysis, Genesky Biotechnologies.Inc., Shanghai, China. The experimental procedure was determined according to a previous study (10) and the experimental timelines are presented in **Figure 8A**. Three groups were created in the experiment: the control, elesclomol-CuCl_2_, and TTM-elesclomol-CuCl_2_ groups. In the elesclomol-CuCl_2_ group, PC9 cells were treated with 30 nM elesclomol-Cu (1:1 ratio) for 2 h. Elesclomol is a potent Cu ionophore that promotes apoptosis. In the TTM-elesclomol-CuCl_2_ group, PC9 cells were pretreated overnight with 20 μM tetrathiomolybdate (TTM), a chelator of copper that acts as an antagonist to cuproptosis, followed by 30 nM elesclomol-Cu (1:1 ratio) pulse treatment for 2 h. The cells in each group were then grown for 48 h in fresh medium before RNA extraction, and qPCR was used to measure gene expression.

### Cell transfection and cell proliferation assays

Small interference RNA (siRNA) targeting *BARX1*, *GFRA3*, and *KHDRBS2* was designed and synthesized from GenePharmal. Sequences for si−BARX1, si−GFRA3, si−KHDRBS2 were listed in **Supplementary Table S3**. Cultured cells with a confluency of 50% were transfected by *BARX1*, *GFRA3*, and *KHDRBS2* specific siRNA using Lipofectamine^®^ 2000 reagent (Invitrogen; Thermo Fisher Scientific, Inc.), with scramble RNA (si–NC) as negative control. After 48 h transfection, cells were collected and were seeded to a 96-well plate at a density of 1 × 10^4^ cells/mL and were treated with different concentration of elesclomol–CuCL_2_ and were cultured with 5% CO2 at 37°C for another 72 h. MTT assays were performed to assess cell proliferation. Specifically, 20 μl MTT (Sigma) was added to each well, and incubated for additional 4 h in the incubator. Then, MTT was removed and 150 μl DMSO was added. After the solvent of purple crystals, the absorbance of the solution at 490 nm was measured as the cell proliferation ability.

### Statistical evaluation

Prism (version 9.0) and R software (version 4.0.3) were used for all statistical analyses. The Kaplan–Meier curves of the two groups of patients were analyzed using the R packages “survival” and “survminer.” Then, using the “survivalROC” R package a time-dependent ROC curve analysis was performed to assess the predictive accuracy of the CRG signatures. The area under the curve (AUC) was used to evaluate prediction ability. *p*-value < 0.05 was considered to be statistically significant, and all *p*-values were two-tailed.

## Results

### Identification of CRGs in TCGA LUAD patients

**Figure 1:**
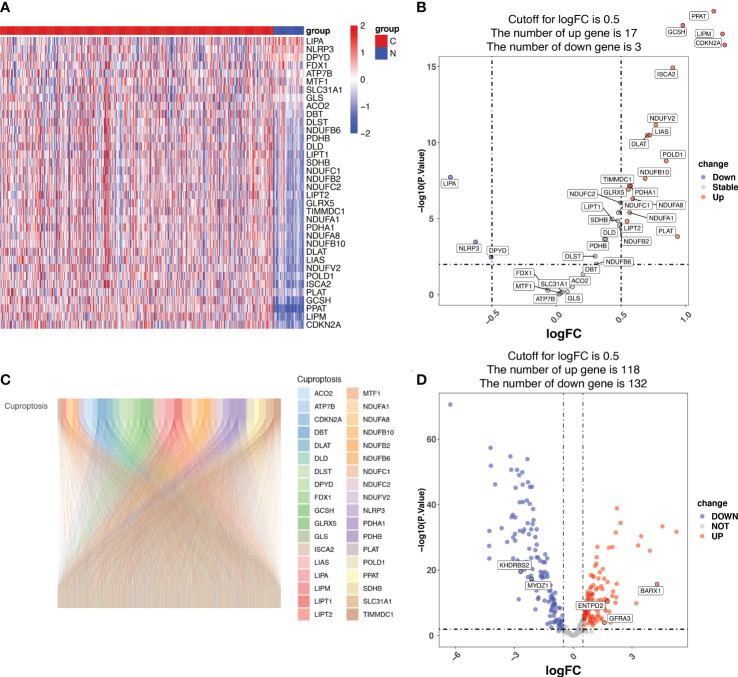
Identification of cuproptosis-related genes (CRGs) in patients with lung adenocarcinoma (LUAD) from the Cancer Genome Atlas (TCGA). **(A)** The heatmap showing the cuproptosis genes in normal and tumor LUAD tissues. N represents normal samples and T represents tumor samples. **(B)** The volcano map displaying cuproptosis genes in the TCGA LUAD cohort. The x-axis represents the log2(Fold Change) (logFC) and the y-axis represents the log10 (*p*-value). The threshold values (logFC>0.5 and *p*-value<0.05) are indicated by dashed lines in the plot and used to classify the genes as ‘unchanged’, ‘downregulated’, or ‘upregulated’. The green dots represent downregulated genes, and the red dots represent upregulated genes. **(C)** The Pearson correlation analysis indicated that 335 CRGs were significantly associated with the expression of cuproptosis genes, which is displayed using the Sankey diagram. **(D)** The volcano map displaying the expression of the 335 CRGs, of which 118 CRGs were upregulated and 132 downregulated in LUAD. The x-axis represents the log2(Fold Change) (logFC) and the y-axis represents the log10 (*p*-value). The threshold values (logFC>0.5 and *p*-value<0.05) are indicated by dashed lines in the plot and used to classify the genes as ‘unchanged’, ‘downregulated’, or ‘upregulated’. The green dots represent downregulated genes, and the red dots represent upregulated genes.

Among the 36 cuproptosis genes from the LUAD samples, 17 were upregulated and 3 were downregulated, indicating dysregulated cuproptosis in LUAD (**Figures 1A, B**). The expression of 335 CRGs was significantly associated with the expression of cuproptosis genes, as revealed by the Pearson correlation analysis (**Supplementary Table S4**). A Sankey diagram demonstrated the CRGs in LUAD (**Figure 1C**). Among the 335 CRGs, 118 were upregulated and 132 were downregulated in LUAD (**Figure 1D**).

### Establishment of cuproptosis-related signature in the training cohort

**Figure 2:**
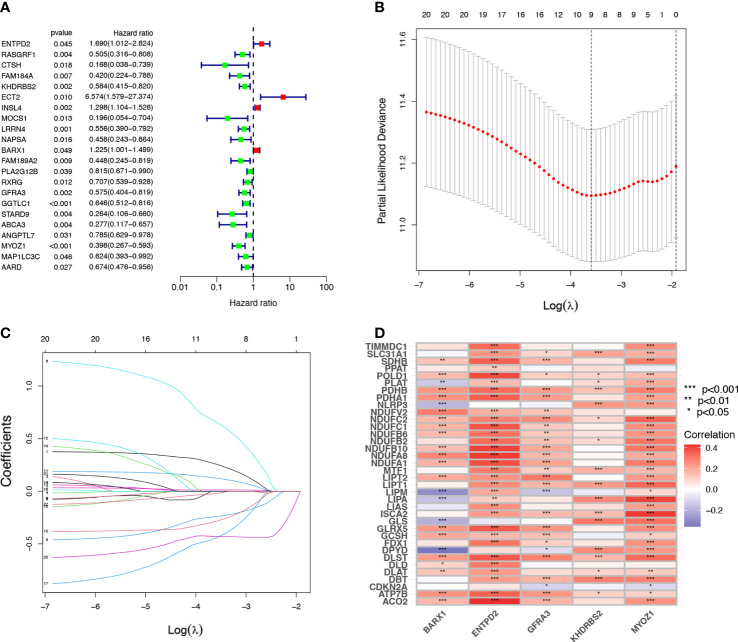
Establishment of cuproptosis-related prognostic signature. **(A)** A univariate Cox regression analysis was used to identify 22 Cuproptosis-related genes (CRGs) with prognostic value. **(B, C)** The 22 CRGs with prognostic value identified using univariate Cox regression analysis were subjected to 1000 iterations of LASSO Cox regression analysis, and finally, five CRGs, including *BARX1*, *ENTPD2*, *GFRA3*, *MYOZ1*, and *KHDRBS2* were filtered to develop a prognostic model. **(D)** The relation of CRGs with the respective prognostic value and their corresponding cuproptosis gene expression are shown in this heatmap *p< 0.05; **p<0.01; ***p<0.001.

**Figure 3:**
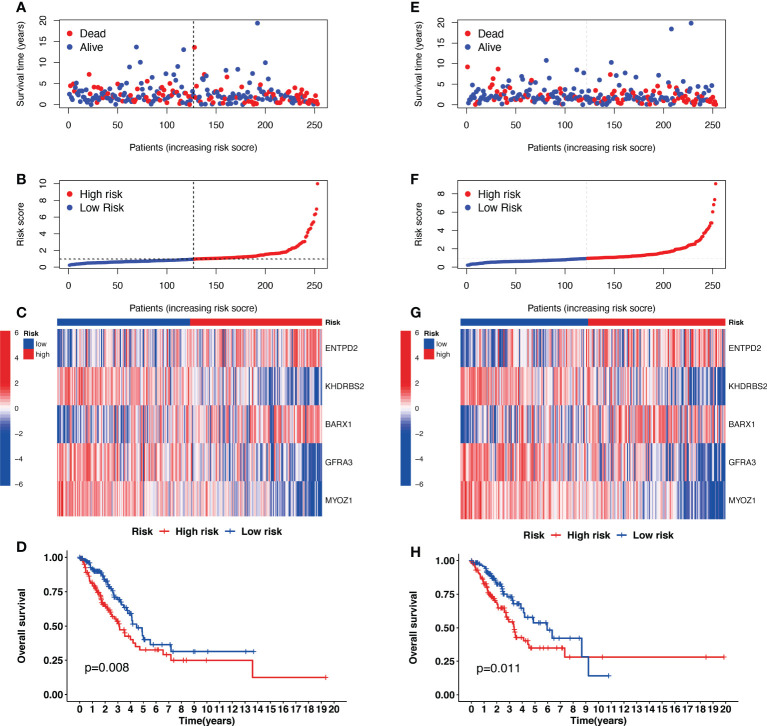
The prognostic analysis of the five-gene signature model in the Cancer Genome Atlas (TCGA) training and testing cohorts. **(A, E)** The risk score distribution and median value in TCGA training and testing cohorts. **(B, F)** The survival status, survival time, and risk score distributions in TCGA training and testing cohorts. **(C, G)** A heatmap of five Cuproptosis-related gene (CRG) gene expression patterns in two risk groups in TCGA training and testing cohorts. **(D, H)** Kaplan–Meier demonstrated the survival of patients in high- or low-risk groups in TCGA training and testing cohorts.

Univariate Cox regression analysis identified 22 prognostic CRGs among the 335 CRGs (**Figure 2A**). The cuproptosis-related signature composed of five CRGs (*BARX1*, *ENTPD2*, *GFRA3*, *KHDRBS2*, and *MYOZ1*) was established using 1000 iterations of LASSO Cox regression analysis (**Figures 2B, C**). The correlation between the Cuproptosis genes and the five prognostic CRGs is presented in the heatmap (**Figure 2D**). The risk score of this signature was calculated as follows: Risk score = (0.2751712 × expr (*ENTPD2*)) + (-0.2280729 × expr (*KHDRBS2*)) + (0.1760958 × expr (*BARX1*)) + (-0.3409875 × expr (*GFRA3*)) + (-0.4459425 × expr (*MYOZ1*)). Based on the median risk score in the TCGA LUAD cohort, all patients were divided into two groups: high-risk (N=126) and low-risk (N=127). Patients in the high-risk group had a higher probability of death than those in the low-risk group (**Figures 3A–C**). In addition, Kaplan–Meier analysis revealed that patients in the high-risk group had significantly shorter OS than those in the low-risk group (**Figure 3D**, *p*=0.008).

### Validation of cuproptosis-related signature in the validation cohort

The risk scores for patients in the validation cohort were calculated to assess the reliability of the five CRG signatures derived from the training cohort. The patients in the TCGA validation cohort were then divided into high-risk (N=126) and low-risk (N=127) groups using the same cutoff value as that of the training cohort. Patients in the high-risk group of the validation cohort had a higher probability of death than those in the low-risk group, similar to that of the training cohort (**Figures 3E–G**). Moreover, Kaplan–Meier survival analysis revealed that patients in the high-risk group had a shorter survival time than those in the low-risk group (**Figure 3H**, *p*=0.011).

**Figure 4:**
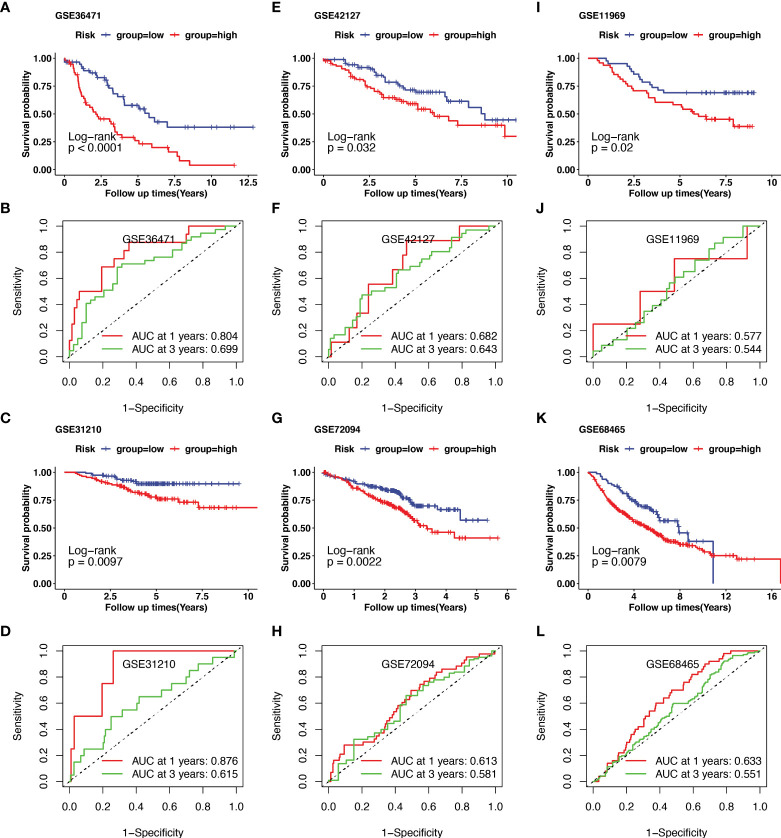
Validation of cuproptosis-related prognostic signature in lung adenocarcinoma (LUAD) from several Gene Expression Omnibus (GEO) cohorts. The external dataset of GSE36471, GSE31210, GSE42127, GSE72094, GSE11969, and GSE68465 were used to validate the prognostic value of the cuproptosis-related genes (CRGs) signature. Kaplan–Meier curve demonstrated the survival of patients in high- or low-risk groups in GSE36471**(A)**, GSE31210**(C)**, GSE42127**(E)**, GSE72094**(G)**, GSE11969**(I)**, and GSE68465**(K)**. The predictive efficacy of 1- and 3-year overall survival in GSE36471**(B)**, GSE31210**(D)**, GSE42127**(F)**, GSE72094**(H)**, GSE11969**(J)**, and GSE68465**(L)** was measured using receiver operating characteristic (ROC) curve.

Additionally, external datasets GSE36471, GSE31210, GSE42127, GSE72094, GSE11969, and GSE68465 were used to validate the prognostic value of the CRG signatures. Kaplan–Meier curves demonstrated that patients in the high-risk group had a worse prognosis than those in the low-risk group in the GSE36471 (N=292, *p*<0.01, **Figure 4A**), GSE31210 (N=158, *p*<0.01, **Figure 4C**), GSE42127 (N=180, *p*=0.03, **Figure 4E**), GSE72094 (N=442, *p*<0.01, **Figure 4G**), GSE11969 (N=90, *p*=0.02, **Figure 4I**), and GSE68465 (N=462, *p*<0.01, **Figure 4K**) cohorts. According to the area under the ROC curve, the predictive efficacy of the CRG signatures for 1- and 3-year OS was 0.804 and 0.699 in GSE36471 (**Figure 4B**), 0.876 and 0.615 in GSE31210 (**Figure 4D**), 0.682 and 0.643 in GSE42127 (**Figure 4F**), 0.613 and 0.581 in GSE72094 (**Figure 4H**), 0.577 and 0.544 in GSE11969 (**Figure 4J**), and 0.633 and 0.551 in GSE68465 (**Figure 4L**) datasets, respectively. These findings showed that the CRG-based prognostic signature had a consistent ability to predict the OS of patients with LUAD.

### The construction of nomogram

**Figure 5:**
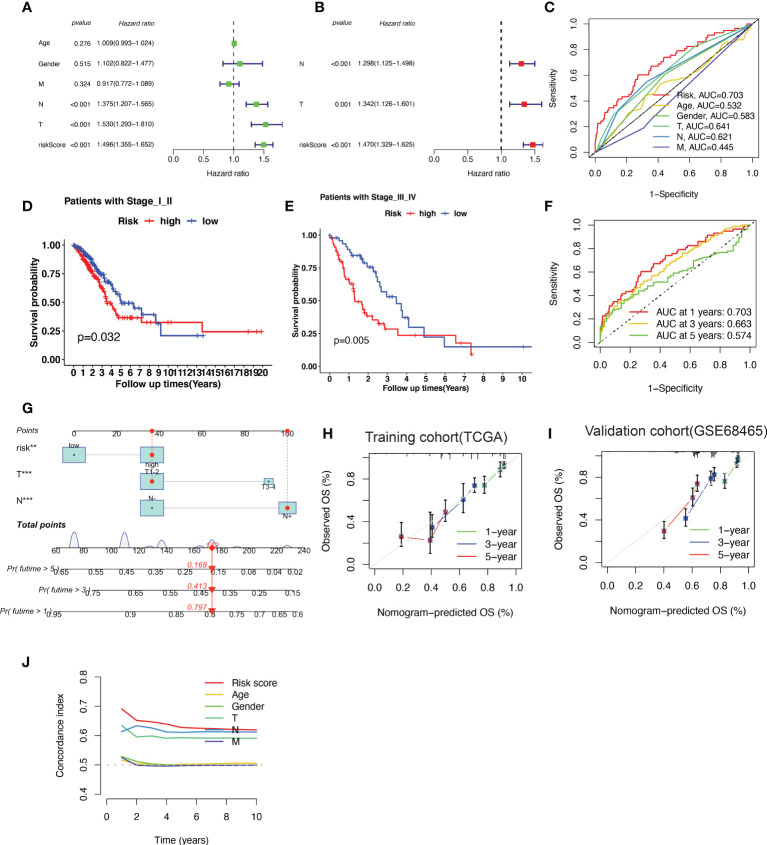
A nomogram predicting the probability of overall survival (OS) based on cuproptosis-related gene (CRG) signatures and clinical factors. **(A, B)** Univariate and Multivariate variable Cox regression analysis of risk factors in lung adenocarcinoma (LUAD). The results indicate that the risk score of the CRG signatures is an independent risk factor. **(C)** area under the curve (AUC) of receiver operating characteristic (ROC) curves used to evaluate the predictive efficacy of the established signature and clinicopathology items for OS. **(D, E)** Patients with different stages (stages I–II and stages III–IV) stratified into high-risk and low-risk groups. Kaplan–Meier curves used to predict the survival of different groups. **(F)** AUC of ROC curves used to evaluate the predictive efficacy of the established signature for the 1-, 3-, and 5-year OS. **(G)** The nomogram predicting the 1-, 3- and 5-year OS constructed by combining the risk score and clinicopathological prognostic indicators. **(H, I)** The calibration chart used to determine the consistency of the nomogram predicted OS and actual OS at 1, 3, and 5 years in both training cohort (TCGA-LUAD) and validation cohort (GSE68465). **(J)** The concordance index (C-index) calculated to measure the nomogram’s capacity to differentiate and predict. The C-index ranges from 0.5–1.0; a higher C-index indicates a stronger differentiating ability.

Based on the results of the univariate analysis, the risk score (HR = 1.496, 95% CI 1.355−1.652), T stage (HR = 1.530, 95% CI 1.293–1.810), and N stage (HR = 1.375, 95% CI 1.207−1.565) were all found to be significantly correlated with the OS of patients with LUAD (**Figure 5A**). To determine whether the risk score acted as an independent prognostic predictor, multivariate Cox regression analyses were used to estimate the risk score and risk factors among clinicopathological items (T stage, N stage). According to multivariate Cox analysis, the risk score (HR = 1.470, 95% CI 1.329–1.625), T stage (HR = 1.342, 95% CI 1.126–1.601) and N stage (HR = 1.298, 95% CI 1.125–1.498) were independent prognostic factors for patients with LUAD (**Figure 5B**). The area under the ROC curve was 0.703 for the risk score, 0.641 for the T stage, and 0.621 for the N stage (**Figure 5C**).

The patients were further categorized into stages I–II and III–IV, according to the NCCN stage. In both stages I–II and III–IV, the patients in the high-risk group had a shorter survival time than those in the low-risk group, as revealed by the Kaplan–Meier survival analysis (**Figure 5D, E**). The area under the ROC curve of the risk factors was 0.703 at 1 year, 0.663 at 2 years, and 0.574 at 5 years (**Figure 5F**).

To better predict 1-, 3- and 5-year survival in patients with LUAD, we combined the data pertaining to the risk score, T stage, and N stage to create a nomogram with a higher total score indicating worse survival (**Figure 5G**). According to the nomogram, the risk score of the CRG-based signature contributed the most to OS in LUAD. The calibration curve showed that the signature for CRGs was highly accurate in both training cohort (TCGA-LUAD) and validation cohort (GSE68465) (**Figure 5H–I**). Furthermore, C-index results indicated that the predictive signature model had the best distinguishing ability (**Figure 5J**).

### Analysis of the connection between risk score, gene mutation status, and survival in LUAD

The somatic mutation information of TCGA-LUAD samples was analyzed and visualized using R package ‘maftools’ (20). Mutations in lung cancer driver genes (*KRAS*, *EGFR*, *BRAF*, *ALK*, *ROS1*, *MET*, *RET*, and *ERBB2*) in the high- and low-risk groups were shown on a waterfall plot, which demonstrated that the low-risk group (20%) harbored a higher *EGFR* mutation frequency than did the high-risk group (8%) (**Supplementary Figure 1A**). The tumor mutation burden was much higher in the high-risk group than that in the low-risk group (**Supplementary Figure 1B**). In addition, survival analysis with subgroups of different *EGFR* statuses was performed to explore the connection between the risk score and EGFR mutation status. The results showed that patients with high-risk factors had reduced survival time both in the EGFR-mutation and EGFR wild-type subgroups (**Supplementary Figures 1C, D**).

### Functional enrichment analysis and tumor immune microenvironment analysis between high- and ow- risk groups

**Figure 6:**
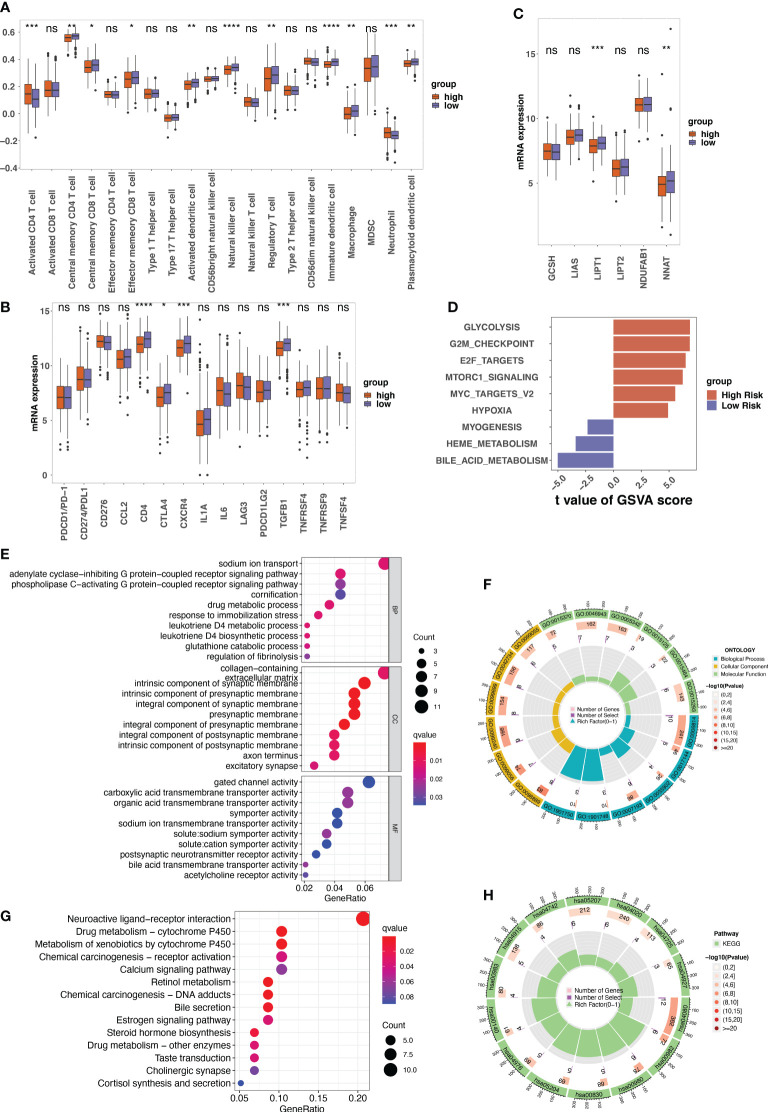
The tumor microenvironment analysis between different risk groups and the annotation results of the differentially expressed genes between risk groups. **(A)** The single sample gene set enrichment analysis (ssGSEA) used to indicate the immune microenvironment in different risk groups. Boxplots showing the comparison of scores of 20 immune cells in the high-risk and the low-risk score groups. **(B)** The comparison between the expression of indicated immune checkpoints in the low- and high-risk groups. **(C)** The expression of indicated genes involved in protein lipoylation compared between low- and high-risk groups. **(D)** The result of gene set variation analysis (GSVA) enrichment in low- and high-risk groups. **(E–H)** The gene ontology (GO) and Kyoto Encyclopedia of Genes and Genomes (KEGG) annotation results of the differentially expressed genes between low- and high-risk groups. ns, not significant, p>0.05; *p<0.05; **p<0.01; ***p<0.001; ****p<0.0001.

The infiltration of immune cells was estimated by “ssGSEA” algorithm. In the low-risk group, we found a higher abundance of immune cells, including central memory CD4+ T cells, central memory CD8+ T cells, effector memory CD8+ T cells, natural killer cells (NK cells), etc. Activated CD4+ T cells and neutrophils were highly enriched in high-risk patients (**Figure 6A**). Additionally, the expression of immune checkpoint inhibitors, particularly CD4, CTLA4, CXCR4, and GFB1, was significantly higher in low-risk patients with LUAD (**Figure 6B**).

Given that cuproptosis is triggered by lipoylated TCA cycle proteins, the protein lipoylation-related genes (*GCSH*, *LIAS*, *LIPT1*, *LIPT2*, *NDUFAB1*, and *NNAT*) among the different risk groups were analyzed. The results revealed that the expression levels of *LIPT1* and *NNAT* were much higher in the low-risk group than in the high-risk group (**Figure 6C**). GSVA analysis of cancer hallmarks revealed that the CRG signature triggered glycolysis, G2M checkpoint, E2F target, and Mtorc1 signaling in the high-risk group. In contrast, the CRG signature in the low-risk group triggered bile acid metabolism, myogenesis, and heme metabolism (**Figure 6D**).

GO annotation of BP revealed that the DEGs between high-risk and low-risk patients were mainly enriched in certain processes such as sodium ion transport and drug metabolic processes. For CC, the DEGs were mainly enriched in collagen-containing extracellular matrix. For MF, gated channel activity and carboxylic acid transmembrane transporter activity were the mainly enriched (**Figures 6E, F**). The KEGG results indicated that these DEGs were involved in neuroactive ligand-receptor interactions, chemical carcinogenesis–DNA adducts, and metabolism of xenobiotics by cytochrome P540 (**Figures 6G, H**).

### Functional enrichment analysis of genes in cuproptosis-related signature

For *BARX1*, KEGG analysis indicated that *BARX1* might be involved in neuroactive ligand-receptor interaction and cAMP signaling pathway (**Supplementary Figure 2A**). GO annotation revealed that the *BARX1* might mainly be enriched in pattern specification process, regionalization, etc. in BP and transmembrane transporter complex, transporter complex, etc. in CC, and channel activity, passive transmembrane transporter activity, etc. in MF (**Supplementary Figure 2B**). The GSVA analysis of cancer hallmarks revealed that the high expression of *BARX1* might trigger unfolded protein response, MYC target, and oxidative phosphorylation, and suppress apoptosis, IL6–JAK–STAT3 signaling, and inflammatory response (**Supplementary Figure 2C**).

For *ENTPD2*, KEGG analysis indicated that *ENTPD2* might be involved in AMPK signaling pathway, cholinergic synapse (**Supplementary Figure 2D**). GO annotation of BP revealed that *ENTPD2* might mainly be enriched in embryonic organ development, cell junction assembly, etc. Whereas for CC, the *ENTPD2* might be enriched in cell-cell junction, apical part of cell, etc., and for MF, metal ion transmembrane transporter activity, anion transmembrane transporter activity, etc. (**Supplementary Figure 2E**). According to the results of the GSVA analysis, *ENTPD2* might activate DNA repair, unfold protein response, and protein secretion while suppressing the inflammatory response and allograft rejection (**Supplementary Figure 2F**).

For *GFRA3*, KEGG analysis indicated that *GFRA3* might also be involved in neuroactive ligand-receptor interaction, calcium signaling pathway, etc. (**Supplementary Figure 2G**). GO annotation of BP revealed that the *GFRA3* might be mainly enriched in pattern specification process, regulation of ion transmembrane transport, etc. Whereas for CC, the *GFRA3* might be enriched in apical part of cell, transmembrane transporter complex, etc., and for MF, passive transmembrane transporter activity, channel activity, etc. (**Supplementary Figure 2H**). The GSVA analysis revealed that high expression of *GFRA3* might trigger the peroxisome, Wnt–β–catenin signaling pathway, and suppress the TNFα signaling *via* NF-κB pathway, and apoptosis (**Supplementary Figure 2I**).

For *KHDRBS2*, KEGG analysis indicated that *KHDRBS2* might be involved in neuroactive ligand-receptor interaction, calcium signaling pathway (**Supplementary Figure 2J**). GO annotation of BP revealed that the *KHDRBS2* might mainly be involved in the regulation of membrane potential, calcium ion homeostasis, etc. In group of CC, the *KHDRBS2* might be enriched in collagen-containing extracellular matrix, transmembrane transporter complex, etc., while in group of MF, passive transmembrane transporter activity, channel activity, etc. (**Supplementary Figure 2K**). The GSVA analysis indicated that *KHDRBS2* might activate the notch signaling pathway and myogenesis, and suppress the glycolysis and MTORC1 signaling (**Supplementary Figure 2L**).

For *MYOZ1*, KEGG analysis showed that *MYOZ1* might be involved in neuroactive ligand-receptor interaction, calcium signaling pathway (**Supplementary Figure 2M**). GO annotation revealed that the *MYOZ1* might be enriched in regulation of ion transmembrane transport, regulation of metal ion transport etc. in BP. Whereas for CC, the *MYOZ1* might be enriched in collagen-containing extracellular matrix and transmembrane transporter complex, etc. and for MF, passive transmembrane transporter activity, channel activity, etc. (**Supplementary Figure 2N**). The GSVA analysis indicated that the high expression of *MYOZ1* might activate the peroxisome, myogenesis, and bile acid metabolism and suppress G2M checkpoint and E2F targets signaling (**Supplementary Figure 2O**).

### Validation of cuproptosis-related signature in specimens and LUAD cells

**Figure 7:**
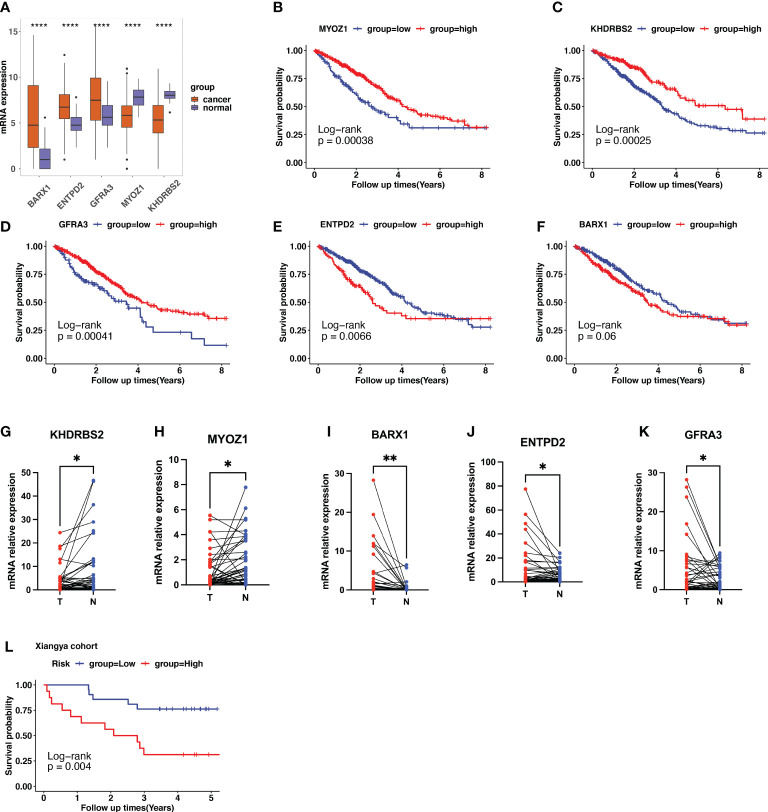
Validation of cuproptosis-related gene (CRG) signatures in clinical specimens. **(A)** The expression patterns of *BARX1*, *ENTPD2*, *GFRA3*, *MYOZ1*, and *KHDRBS2* in the Cancer Genome Atlas (TCGA) lung adenocarcinoma (LUAD) cohort. **(B–F)** In TCGA LUAD dataset, higher expression of *MYOZ1*, *KHDRBS2*, and *GFRA3* indicates longer survival times using the Kaplan–Meier curve with the log-rank test. In contrast, higher expression of *ENTPD2* and *BARX1* indicates a shorter survival period. **(G–K)** The expression level of the five prognostic CRGs further validated using the 37 clinical LUAD samples. The expression of *KHDRBS2* and *MYOZ1* was downregulated in the LUAD cancer samples compared with that of para-tumor normal samples, whereas the expression of *BARX1*, *ENTPD2*, and *GFRA3* was upregulated in LUAD cancer tissues. The scatter plots depicting the expression patterns. N represents normal samples and T represents tumor samples. **(L)** Patients in our clinical cohorts were divided into high-risk and low-risk groups based on the risk score, which was calculated using the risk formula mentioned above. The survival analysis using Kaplan–Meier survival curve showed that patients in the high-risk group have a short overall survival compared with those in the low-risk group. *p<0.05; **p<0.01; ****p<0.0001.

In TCGA LUAD dataset, the expression of *KHDRBS2*, *MYOZ1*, *BARX1*, *ENTPD2*, and *GFRA3* are displayed in **Figure 7A**. When compared with normal samples, tumor samples showed higher expression of *BARX1*, *ENTPD2*, and *GFRA3*, and lower expression of *MYOZ1* and *KHDRBS2*. The Kaplan–Meier curve with the log-rank test showed that high expression of *MYOZ1*, *KHDRBS2*, and *GFRA3* indicated a better clinical outcome (**Figures 7B–D**). The higher expression of *BARX1* and *ENTPD2* indicated shorter OS (**Figures 7E, F**).

Furthermore, we performed qPCR to validate the expression patterns of the five prognostic genes in 37 clinical specimens. The expression of *KHDRBS2* and *MYOZ1* was downregulated in the LUAD cancer samples compared with that in the para-tumor normal samples (**Figures 7G, H**), whereas the expression of *BARX1*, *ENTPD2*, and *GFRA3* was upregulated in LUAD cancer tissues (**Figures 7I–K**). Risk score was calculated using the formula described. Patients in the clinical cohort were divided into high- and low-risk groups based on their risk scores. Survival analysis showed that patients with a higher risk score had a shorter OS than those with a lower risk score (**Figure 7L**, *p*=0.004).

### The *BARX1* and *GFRA3* deficiency sensitize the LUAD cells to cuproptosis inducer

**Figure 8:**
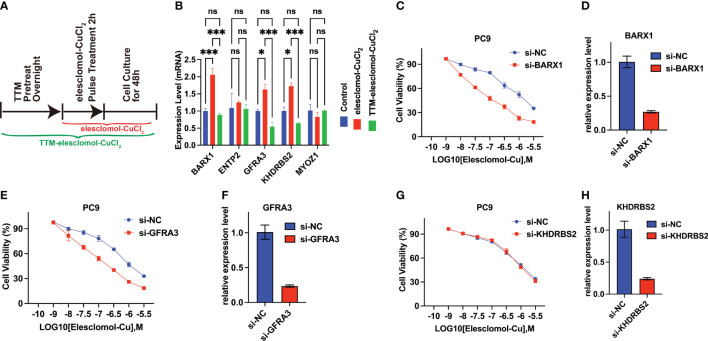
*BARX1* and *GFRA3* are related to copper ionophore-induced cell death. **(A)** *In vitro* experiment was performed to investigate the relationship between cuproptosis and the expression of CRGs. The experiment was conducted using three different groups: control, elesclomol-CuCl_2_, and TTM-elesclomol-CuCl_2_. Each group in the experiment followed the indicated timelines that were provided. In the elesclomol-CuCl_2_ group, PC9 cells were treated with 30 nM elesclomol-CuCl_2_ (1:1 ratio) by pulsing; in the TTM-elesclomol-CuCl_2_ group, PC9 cells were pretreated overnight with 20 mM TTM, a chelation of copper that inhibits cuproptosis, followed by pulse treatment with 30 nM elesclomol-CuCl_2_ (1:1 ratio). **(B)** The gene expression was tested by real-time quantitative PCR (qPCR) 48 hours after indicted treatments (fold change over control). ns, not significant, *p*>0.05; *p< 0.05; ***p<0.001. **(C–H)** PC9 cells transfected with *BARX1***(C)**, *GFRA3* **(E)**, *or KHDRBS2***(G)** specific small interfering RNA (siRNA) were analyzed for viability 72 hours after treatment of indicated concentration of elesclomol-CuCl_2,_ with the scramble siRNA as the control(si-NC). The knockdown efficiency of *BARX1***(D)**, *GFRA3***(F)**, and *KHDRBS2***(H)** was indicated by the qPCR.

A cuproptosis induction model was constructed to explore whether the expression of *ENTPD2*, *KHDRBS2*, *BARX1*, *GFRA3*, and *MYOZ1* was affected by cuproptosis. The results revealed that the expression of *BARX1*, *GFRA3*, and *KHDRBS2* was upregulated after cuproptosis was induced by elesclomol-CuCL_2_, whereas the upregulation was suppressed when PC-9 cells were pretreated with TTM (**Figure 8B**). In contrast, the expression of *NETP2* and *MYOZ1* remained intact in both the elesclomol-CuCl_2_ and TTM-elesclomol-CuCl_2_ groups compared with that of the control group (**Figure 8B**).

In addition, to explore the functions of the *BARX1*, *GFRA3*, and *KHDRBS2* in cuproptosis in LUAD, the sensitivity of LUAD cells to cuproptosis was determined after the knockdown of target genes. As indicated by the qPCR, the efficiency of *BARX1* (**Figure 8D**), *GFRA3* (**Figure 8F**), and *KHDRBS2* (**Figure 8H**) silencing in the PC9 cells was satisfied after the 48 h transfection of siRNA. The cell proliferation assay revealed that the knockdown of BARX1(**Figure 8C**) and *GFRA3*(**Figure 8E**) deficiency facilitated the cuproptosis induced by elesclomol-CuCl_2_, whereas the sensitivity of PC9 cells to cuproptosis inducers remains intact after the knockdown of *KHDRBS2* (**Figure 8G**).

## Discussion

Cell death is the end of the cell life cycle and is critical for the survival and development of an organism. Necrosis and apoptosis are the two most prevalent types of cell death (21). Other types of cell death have recently been discovered, including autophagy (22), necroptosis (22), pyroptosis (23), and ferroptosis (24). More recently, Tsvetkov et al. presented cuproptosis, which is caused by direct binding of Cu to the lipoylated components of the TCA cycle, leading to lipoylated protein aggregation. The subsequent loss of Fe-S cluster proteins results in proteotoxic stress and ultimately cell death (10). Specifically, Cu increased mitochondrial protein lipoylation, a post-translational modification of lysine that occurs in four enzymes that regulate carbon entry into the TCA cycle (dihydrolipoamide branched chain transacylase E2 (*DBT*), glycine cleavage system protein H (*GCSH*), dihydrolipoamide S-succinyltransferase (*DLST*), and dihydrolipoamide S-acetyltransferase (*DLAT*)). Cu binds directly to *DLAT*, enhancing lipoylated *DLAT* aggregation *via* disulfide bonds. Ferredoxin 1 (*FDX1*), in particular, is a recently discovered lipoylation effector that leads to the accumulation of toxic lipoylated *DLAT*, consequently leading to cuproptosis. We found that the expression of *GCSH* and *DLAT* was much higher in tumor tissues than in normal tissues in LUAD (**Figure 1B**). Thus, the cuproptosis pathway is expected to be a promising new target in lung cancer treatment.

However, there has been very little research on CRGs in LUAD. Therefore, Pearson correlation analysis was performed to identify CRGs, and the prognostic value of the CRGs was investigated. We identified 22 CRGs that were significantly related to the overall survival of patients with LUAD. In addition, based on their performance in the LASSO Cox regression analysis, five CRGs (*ENTPD2*, *KHDRBS2*, *BARX1*, *GFRA3*, and *MYOZ1*) were chosen to construct a prognostic signature. Moreover, an outcome risk nomogram was created using the gene signature and clinicopathological characteristics to quantify the outcome risk for each patient. Independent datasets (GSE36471, GSE31210, GSE42127, GSE72094, GSE11969, and GSE68465) were used to validate the prognostic value of this signature. The results of the time–ROC and Kaplan–Meier survival curves confirmed that this signature could be a more effective predictor of patient prognosis.

The qPCR results of the clinical samples were consistent with those obtained from the datasets. Specifically, the expression of *KHDRBS2* and *MYOZ1* was downregulated in LUAD tumor samples. *KHDRBS2* is speculated to be an RNA-binding protein that affects mRNA splice site selection and exon inclusion in addition to playing a role in alternative splicing control. However, its role in cancer and cuproptosis remains unclear. The protein produced by *MYOZ1* belongs to the myozenin family and is predominantly expressed in the skeletal muscle. Nonetheless, its role in tumorigenesis remains unclear. The expression levels of *ENTPD2*, *BARX1*, and *GFRA3* were higher in LUAD tumor samples than in normal para-tumor samples. Hypoxia stimulates the expression of *ENTPD2* in cancer cells, resulting in increased extracellular 5’-AMP, which promotes the maintenance of myeloid-derived suppressor cells (MDSCs) by blocking their differentiation in hepatocellular carcinoma and leading to escape of cancer cells from immune surveillance (25). Inhibition of *ENTPD2* suppresses the formation and migration of lung adenocarcinoma cells (26). *BARX1* encodes a transcription factor linked to poor prognosis and may promote clear cell renal cell carcinoma (ccRCC) proliferation and migration (27). In gastrointestinal stromal tumors, *BARX1* acts as a transcriptional and anatomical determinant of malignancy (28). *GFRA3* may be involved in the RET pathway. Methylation of the *GFRA3* promoter has been linked to worse postoperative survival rates in patients with gastric cancer (29). Similarly, in our study, low expression of *GFRA3* in LUAD was associated with worse survival. Finally, the higher risk score based on the expression patterns of these five genes indicated a worse survival rate in our clinical results. Further *in vitro* experiments revealed that the expression of *BARX1*, *GFRA3*, and *KHDRBS2* can be upregulated after induction of cuproptosis by elesclomol-CuCL_2_. However, the upregulation of these genes was suppressed when the cells were pretreated with TTM. This suggests that these genes are closely linked to cuproptosis. Further, cell proliferation assay revealed that the silencing of *BARX1* and *GFRA3* increased the sensitivity of LUAD cells to cuproptosis. Therefore, targeting *BARX1* and *GFRA3* could be a strategy for designing and producing the sensitizers of cuproptosis inducer. Nevertheless, more research is needed to establish the mechanism by which these genes regulate apoptosis.

GSVA analysis showed that the glycolysis pathway was enriched in high-risk patients. Tsvetkov et al. reported that Cu ionophore-induced cell death was regulated by mitochondrial respiration. Cells that rely on mitochondrial respiration are more sensitive to Cu ionophores than the cells that rely on glycolysis. Glycolysis is crucial for cancer cell growth Thus, inhibiting glucose metabolism would, reduce the malignant potential of these cells in addition to making them more susceptible to therapy with Cu ionophores. These results may also indicate greater sensitivity to treatment with Cu ionophores in the low-risk groups.

Cuproptosis is triggered by lipoylated TCA cycle proteins. Thus, we explored the expression of protein lipoylation-related genes (*GCSH*, *LIAS*, *LIPT1*, *LIPT2*, *NDUFAB1*, and *NNAT*) between the low- and high-risk groups. Expression of *LIPT1* and *NNAT* was significantly higher in the low-risk group than in the high-risk group. Tsvetkov et al. found that the killing effect of Cu ionophores was attenuated by knockout of lipolytransferase1 (*LIPT1*). This suggests that *LIPT1* may also play a critical role in the specific metabolic pathways that mediate copper toxicity. A higher expression level of *LIPT1* in the low-risk group may indicate a higher sensitivity to the Cu ionophore.

The immune microenvironment is closely linked to various types of cell death such as ferroptosis (30) and apoptosis (31). However, the relationship between cuproptosis and immune cell infiltration in LUAD remains unknown. The ssGSEA algorithm was used to calculate the proportion of different types of tumor-infiltrating immune cells. The findings revealed that when compared with the high-risk group, greater infiltration of immune cells, including central memory CD4+ T cells, central memory CD8+ T cells, effector memory CD8+ T cells, and natural killer cells (NK cells), was observed in the low-risk group. Furthermore, the group classified as high-risk based on the cuproptosis-related signature in LUAD tended to have lower expression levels of immune checkpoint molecules, including *CD4*, *CTLA4*, *CXCR4*, and *TGFB1*. Immunologically, the tumors can be separated into “hot” and “cold” tumors. The accumulation of proinflammatory cytokines and increased T cell infiltration in “hot” tumors are indicators of their propensity to respond well to ICIs. Cold tumors, on the other hand, have less T cell infiltration and proinflammatory cytokine production and may respond less well to ICB therapy (32, 33).The results showed that LUAD cells in the high-risk group tended to be immunologically “cold,” making them resistant to the immune checkpoint inhibitors, whereas based on cuproptosis-related signature in the low-risk group, LUAD cells tended to be immunologically “hot,” making them more likely to benefit from immune checkpoint inhibitors.

Our study had a few limitations. First, our research was largely based on public datasets, and only a small number of clinical samples from a single center were used to retrospectively validate the prognostic value of the cuproptosis-related signature. This prognostic model remains to be confirmed using prospective multicenter real-world data. The *in vitro* experiment identified only a preliminary link between CRGs and cuproptosis. Furthermore, the mechanism underlying cuproptosis regulation by CRGs requires further investigation. Further research is needed to identify the pathways through which these CRGs are implicated in the carcinogenesis of lung cancer.

## Data availability statement

The datasets presented in this study can be found in online repositories. The names of the repository/repositories and accession number(s) can be found in the article/**Supplementary Material**.

## Ethics statement

The studies involving human participants were reviewed and approved by the institutional ethics committee of Xiangya Hospital of Central South University. The patients/participants provided their written informed consent to participate in this study.

## Author contributions

The study was conceived and designed by LL and YC. YC and LL analyze the data. The manuscript was written by YC, LT, and FA. The experiment was conducted by YC, YZ, and WH. The manuscript was revised by YC and LL. All authors contributed to the article and approved the submitted version.

## Funding

This work was supported by Natural Science Foundation of Hunan Province (2022JJ30995).

## Conflict of interest

The authors declare that the research was conducted in the absence of any commercial or financial relationships that could be construed as a potential conflict of interest.

## Publisher’s note

All claims expressed in this article are solely those of the authors and do not necessarily represent those of their affiliated organizations, or those of the publisher, the editors and the reviewers. Any product that may be evaluated in this article, or claim that may be made by its manufacturer, is not guaranteed or endorsed by the publisher.

## Supplementary material

Supplementary Figure 1
**(A)** The waterfall plot presenting the gene mutations between high- and low-risk groups. **(B)** The tumor mutation burden is much higher in a high-risk group than in the low-risk group. **(C, D)** The survival analysis performed between subgroups with different *EGFR* statuses (*EGFR* mutation and *EGFR* wild-type). Kaplan–Meier curve analysis revealed that survival was worse in patients with high-risk scores in both subgroups.

Supplementary Figure 2 The function of the *BARX1*, *ENTPD2*, *GFRA3*, *KHDRBS2*, and *MYOZ1* was explored by the GO annotation, KEGG, and GSVA analysis. **(A–C)** The KEGG, GO, and GSVA analysis of *BARX1*. **(D–F)** The KEGG, GO, and GSVA analysis of *ENTPD2*. **(G–I)** The KEGG, GO, and GSVA analysis of *GFRA3*. **(J–L)** The KEGG, GO, and GSVA analysis of *KHDRBS2*. **(M–O)** The KEGG, GO, and GSVA analysis of *MYOZ1*.
