# Supplementary figures and images for: Identification and validation of a novel cuproptosis-related signature as a prognostic model for lung adenocarcinoma
Source: Front Endocrinol (Lausanne). 2022 Oct 24;13:963220. doi: 10.3389/fendo.2022.963220 (PMC9637654; doi:10.3389/fendo.2022.963220)

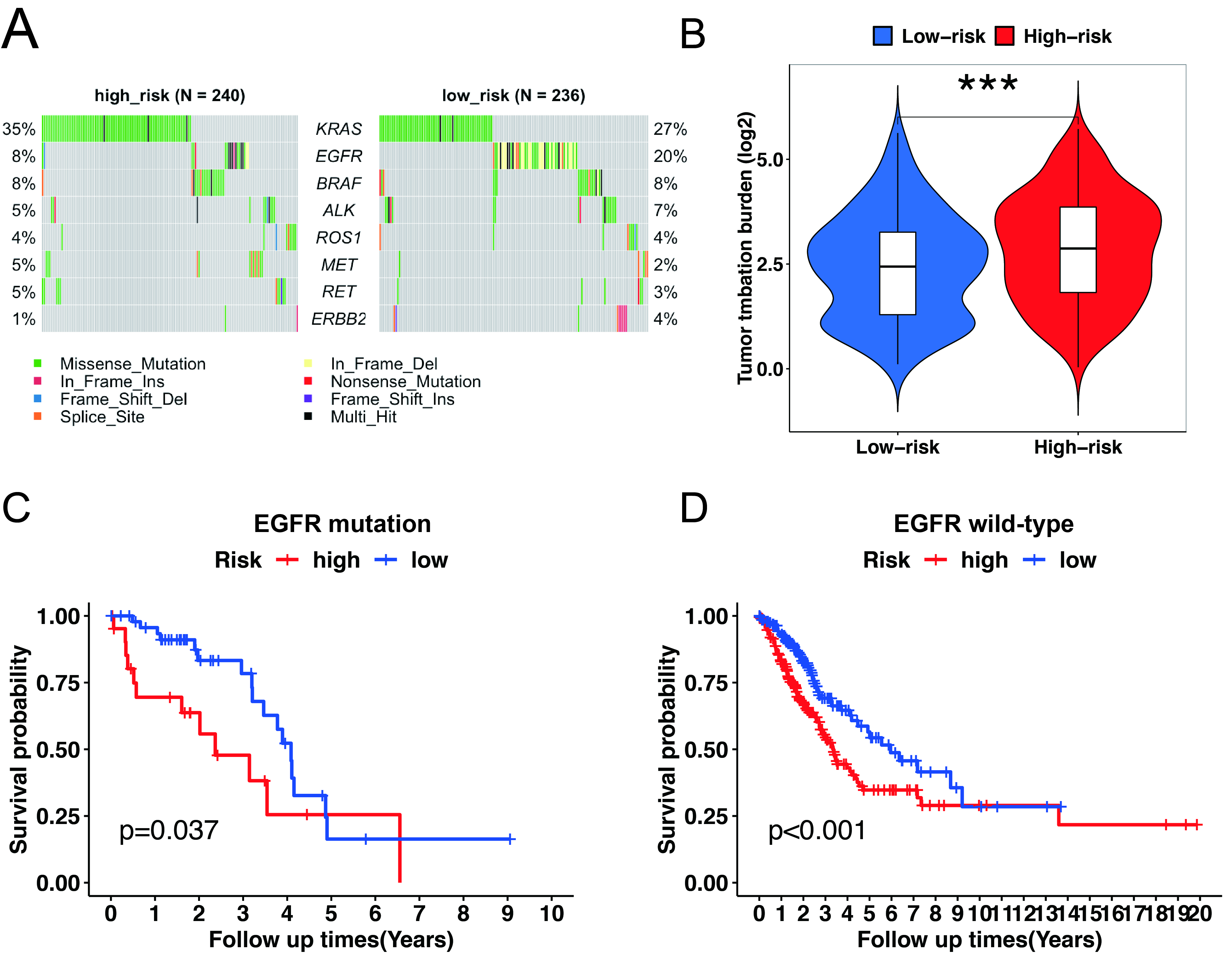

Supplement: Supplementary Figure 1 — (A) The waterfall plot presenting the gene mutations between high- and low-risk groups. (B) The tumor mutation burden is much higher in a high-risk group than in the low-risk group. (C, D) The survival analysis performed between subgroups with different EGFR statuses (EGFR mutation and EGFR wild-type). Kaplan–Meier curve analysis revealed that survival was worse in patients with high-risk scores in both subgroups. [file Image_1.tif]

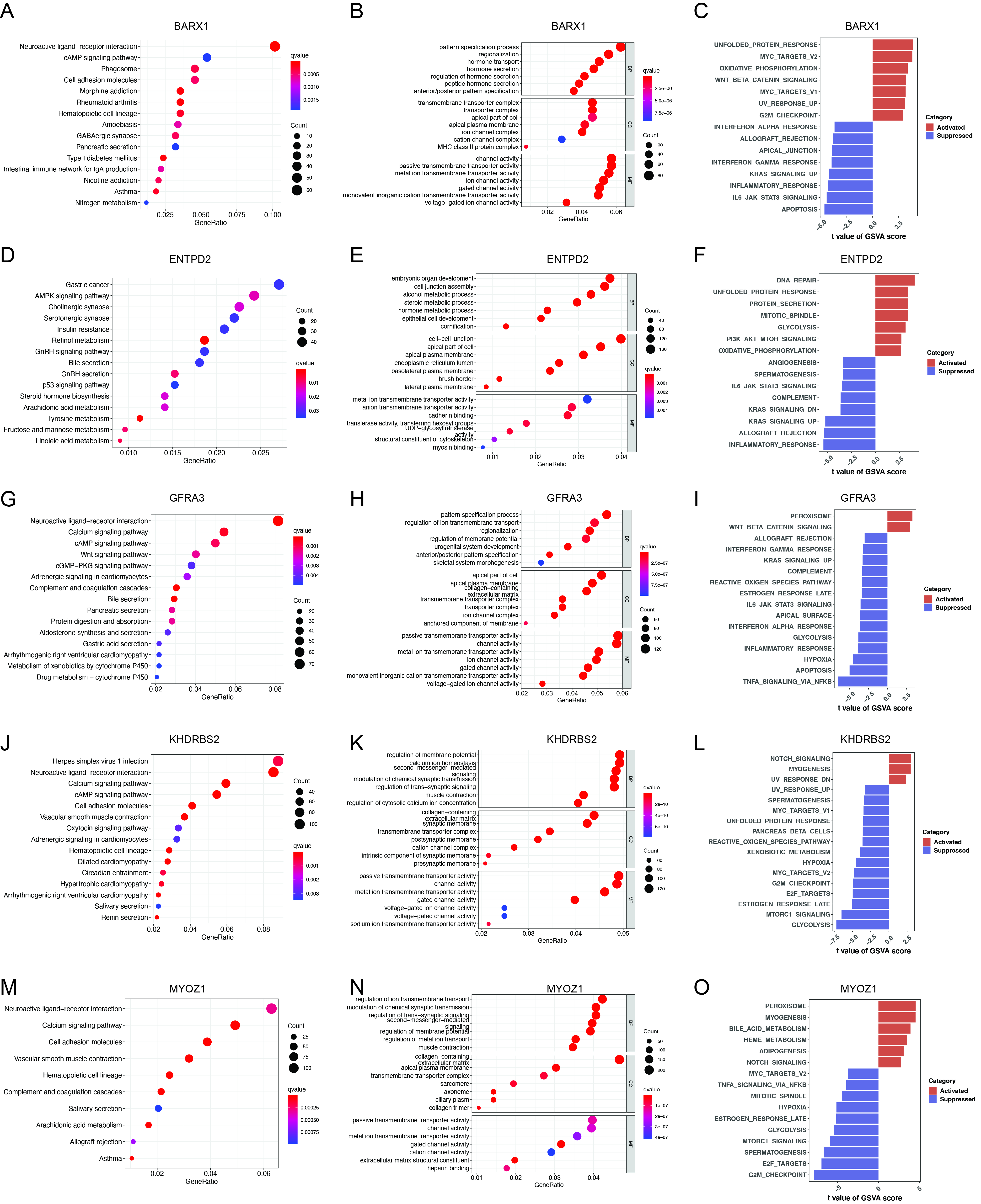

Supplement: Supplementary Figure 2 — The function of the BARX1, ENTPD2, GFRA3, KHDRBS2, and MYOZ1 was explored by the GO annotation, KEGG, and GSVA analysis. (A–C) The KEGG, GO, and GSVA analysis of BARX1. (D–F) The KEGG, GO, and GSVA analysis of ENTPD2. (G–I) The KEGG, GO, and GSVA analysis of GFRA3. (J–L) The KEGG, GO, and GSVA analysis of KHDRBS2. (M–O) The KEGG, GO, and GSVA analysis of MYOZ1. [file Image_2.tif]
