# Supplementary material for: Identification and validation of a novel cuproptosis-related signature as a prognostic model for lung adenocarcinoma
Source: Front Endocrinol (Lausanne). 2022 Oct 24;13:963220. doi: 10.3389/fendo.2022.963220 (PMC9637654; doi:10.3389/fendo.2022.963220)
Supplement: Supplementary file 4 [file Table_2.docx]

| The oligonucleotides information | |
| --- | --- |
| F_ACTB | GATTCCTATGTGGGCGACGA |
| R_ACTB | AGGTCTCAAACATGATCTGGGT |
| F_ENTPD2 | GACCTGGGCTCAGCAGC |
| R_ENTPD2 | AAATGGTGCTTGGCAGCTTG |
| F_KHDRBS2 | GGTGGCAAAATCTCACCAGG |
| R_KHDRBS2 | TTCCCTGTATCCCCCTCTGG |
| F_BARX1 | TTCCACGCCGGACAGAATAG |
| R_BARX1 | CTGCTCGCTCGTTGGAATTG |
| F_GFRA3 | AATGACAAGTGTGACCGGCT |
| R_GFRA3 | ACCAGGCGTGATCTGCAAAG |
| F_MYOZ1 | GTGAGACAGGATCAGGAGACC |
| R_MYOZ1 | GAAGGCCTGTTGGAGAGGTT |

Supplementary TableS2: The primer sequences used in this paper
