# Supplementary material for: Identification and validation of a novel cuproptosis-related signature as a prognostic model for lung adenocarcinoma
Source: Front Endocrinol (Lausanne). 2022 Oct 24;13:963220. doi: 10.3389/fendo.2022.963220 (PMC9637654; doi:10.3389/fendo.2022.963220)
Supplement: Supplementary file 5 [file Table_3.docx]

| The oligonucleotides information | |
| --- | --- |
| si−*BARX1*–1#–sense | GCCUGGAGAAACGCUUCGATT |
| si−*BARX1*–1#–antisense | UCGAAGCGUUUCUCCAGGCTT |
| si−*BARX1*–2#–sense | CCACGCCGGACAGAAUAGATT |
| si−*BARX1*–2#–antisense | UCUAUUCUGUCCGGCGUGGTT |
| si−*GFRA3*–1#–sense | GCCUGCUUGGACAUCUAUUTT |
| si−*GFRA3*–1#–antisense | AAUAGAUGUCCAAGCAGGCTT |
| si−*GFRA3*–2#–sense | GCUGCUCACUUUCUUCGAGTT |
| si−*GFRA3*–2#–antisense | CUCGAAGAAAGUGAGCAGCTT |
| si−*KHDRBS2*–1#–sense | GGGAAAUUGCUUGGACCAATT |
| si−*KHDRBS2*–1#–antisense | UUGGUCCAAGCAAUUUCCCTT |
| si−*KHDRBS2*–2#–sense | GACCAGACCUAUGAGACUUTT |
| si−*KHDRBS2*–2#–antisense | AAGUCUCAUAGGUCUGGUCTT |

Supplementary TableS3: The specific small interfering RNA sequences (siRNA) of *BARX1*, *GFRA3*, and *KHDRBS2* used in this study.
